# Enhancement of SARS-CoV-2 receptor-binding domain activity by two microbial defensins

**DOI:** 10.3389/fmicb.2023.1195156

**Published:** 2023-06-19

**Authors:** Bin Gao, Shunyi Zhu

**Affiliations:** Group of Peptide Biology and Evolution, State Key Laboratory of Integrated Management of Pest Insects and Rodents, Institute of Zoology, Chinese Academy of Sciences, Beijing, China

**Keywords:** AMSIN, positive allosteric peptide, protein binder, coronavirus, ACE2

## Abstract

Peptide binders are of great interest to both basic and biomedical research due to their unique properties in manipulating protein functions in a precise spatial and temporal manner. The receptor-binding domain (RBD) of the SARS-CoV-2 Spike protein is a ligand that captures human angiotensin-converting enzyme 2 (ACE2) to initiate infection. The development of binders of RBDs has value either as antiviral leads or as versatile tools to study the functional properties of RBDs dependent on their binding positions on the RBDs. In this study, we report two microbe-derived antibacterial defensins with RBD-binding activity. These two naturally occurring binders bind wild-type RBD (WT RBD) and RBDs from various variants with moderate-to-high affinity (7.6–1,450 nM) and act as activators that enhance the ACE2-binding activity of RBDs. Using a computational approach, we mapped an allosteric pathway in WT RBD that connects its ACE2-binding sites to other distal regions. The latter is targeted by the defensins, in which a cation-π interaction could trigger the peptide-elicited allostery in RBDs. The discovery of the two positive allosteric peptides of SARS-CoV-2 RBD will promote the development of new molecular tools for investigating the biochemical mechanisms of RBD allostery.

## Introduction

The development of binders of proteins is an emerging field. These molecules can bind to specific proteins to block or perturb protein function and thus offer a new, direct way to study protein–protein interactions and to assess protein function, distribution, and dynamics *in vivo* (Bieli et al., 2016). The application of protein binders has made developmental biology research greatly beneficial in that they enable protein function to be regulated, both systematically and in a precise spatial and temporal manner (Harmansa and Affolter, 2018). For the model organism *Drosophila*, binders are also used to directly and acutely manipulate proteins for investigating development and homeostasis (Lepeta et al., 2022). In the field of biological technology, protein binders have proven useful in affinity purification of proteins of interest (Heu et al., 2014). More importantly, some recently developed genetically encoded protein binders have shown prospects for biomedical applications (Bonadio and Shifman, 2021).

Because ligand binding can change the structure and function of a distal functional region of proteins, binders are also a useful tool to study protein allostery, a natural phenomenon in proteins whereby distal structural elements are dynamically coupled (Dokholyan, 2016). The Spike (S) protein of severe acute respiratory syndrome coronavirus 2 (SARS-CoV-2) (Arya et al., 2021) is a specialized ligand that mediates viral entry into a cell to initiate infection via the C-terminal domain of the S protein (known as receptor-binding domain, RBD) to attach to the host receptor—angiotensin-converting enzyme 2 (ACE2) (Kuhn et al., 2004; Shang et al., 2020). Exploring the binders specifically targeting the RBD is of great value either as an antiviral agent candidate if this binder can inhibit the RBD-ACE2 interaction or as a tool to study the RBD function *in vitro* and *in vivo* and allostery related to viral infection if the binder interacts with a non-ACE2-binding surface in RBD. Employing a phage biopanning technique, Yu et al. (2022) identified a peptide binder R1 from a phage-displayed peptide library, which shows a high affinity for the SARS-CoV-2 Spike RBD. Using the microscale thermophoresis (MST) technique, Gao and Zhu identified a fungal defensin (micasin) that targets the SARS-CoV-2 Spike RBD (Gao and Zhu, 2021).

In this study, we report two new, naturally occurring binders that can bind a series of SARS-CoV-2 RBD proteins with moderate-to-high affinity. There are two microbial defensins [namely AMSIN from actinomyces (Zhu et al., 2022) and emorisin from a fungus identified here], which both work as activators of RBDs via allostery. Despite different origins, they both share a conserved structural motif that is involved in interactions with RBDs. A cation-π interaction was proposed to trigger the peptide-elicited allostery in RBD. In addition to the value in basic research, these two binders could also be useful as diagnostic agents for antigen detection of SARS-CoV-2 in human serum.

## Materials and methods

### Gene discovery of emorisin

Emorisin was discovered by using TBLASTN (protein sequence searched against translated nucleotide sequences) searching the fungal genomic sequences deposited in the GenBank database (https://www.ncbi.nlm.nih.gov/) using micasin, a dermatophytic defensin (Zhu et al., 2012), as a query.

### Structural modeling

The structure of emorisin was built by trRosetta (transform-restrained Rosetta) (Du et al., 2021), a server for fast and accurate protein structure prediction, powered by deep learning and Rosetta (https://yanglab.nankai.edu.cn/trRosetta/). The confidence of the model built with restraints from both deep learning and the homologous templates is very high with an estimated TM score of 0.735.

### Molecular docking

The HEX program (version 8.0.0) (http://hex.loria.fr/) that uses a fast Fourier transform (FFT)-based rigid-body docking algorithm to perform protein–protein docking was used to establish initial complexes between AMSIN (PDB entry 2RU0) and WT RBD (PDB entry 6LZG). A set of top complexes (128 poses) were first generated based on shape complementation, and then, the poses with the N-terminal Gly–Phe–Gly residues of AMSIN and the ACE2-binding sites (ABSs) of WT RBD not implicated in binding were chosen based on experimental data. Subsequently, we carried out the second round of filtering to choose the poses with WT RBD residues relevant to the ABSs directly interacting with AMSIN. The final docking pose chosen was used for energy minimization using the force field of AMBER 14 implemented in Molecular Operating Environment (MOE) 2019.0102 (https://www.chemcomp.com/index.htm) and further structural analysis.

### Prediction of cation-π interactions

Intermolecular cation-π interactions in the AMSIN-WT RBD complex were predicted with the CaPTURE program written by Justin Gallivan (Gallivan and Dougherty, 1999) (http://capture.caltech.edu/), in which E_es_ and E_vdW_ represent electrostatic and van der Waals interactions, respectively.

### Residue relevance analysis

ProteinLens (https://www.proteinlens.io/webserver/), an atomistic graph-theoretical method for the investigation of allosteric signaling within one molecule (Mersmann et al., 2021), was used to analyze potential allosteric residues relevant to ABSs in WT RBD and its variants. The PDB entries used were 7FEM (αRBD), 7EKG (βRBD), 7EKC (γRBD), 7WBQ (δRBD), and 7WBP (oRBD). ABSs in these RBDs were selected as source sites for ProteinLens analysis (Gu et al., 2022).

### Oxidative refolding of chemically synthesized peptides

Emorisin and (ΔGFG)AMSIN, a mutant of AMSIN (Zhu et al., 2022) with the N-terminal Gly–Phe–Gly residues deleted, were chemically synthesized in ChinaPeptides Co., Ltd. (Shanghai, China) with >95% purity. For oxidative refolding of emorisin, the synthetic peptide was dissolved in water at a concentration of 2 mg/ml, and then, 100 mM Tris-HCl (pH 8.5) was used to dilute the peptide solution to a final concentration of 0.1 mg/ml. The solution was incubated at 25°C for 48 h, and the oxidized product was then purified by reversed-phase high-performance liquid chromatography (RP-HPLC). For (ΔGFG)AMSIN, the peptide was dissolved in water at a concentration of 2 mg/ml, and then, dimethyl sulfoxide (DMSO) was added to a final concentration of 10% (v/v). The mixture was incubated at room temperature for 30 min, and then, 0.8 ml of PBS buffer (140 mM NaCl, 2.7 mM KCl, 10 mM Na_2_HPO4, and 1.8 mM KH_2_PO4, and pH 7.5) was added. After incubating overnight at 25°C, the cyclic product was purified by RP-HPLC. The collected peaks were lyophilized by Thermo Scientific SAVANT SPD1010 SpeedVac Concentrator (Waltham, MA, USA), and their purity and molecular weights were identified by matrix-assisted laser desorption/ionization time-of-flight mass spectrometry (MALDI-TOF) using an UltrafleXtreme^TM^ instrument (Bruker Daltonics, Bremen, Germany) in the positive-ion mode and α-cyano-4-hydroxycinnamic acid (CHCA) as a liquid matrix.

### Circular dichroism spectroscopy

For CD analysis, a peptide sample (emorisin or (ΔGFG)AMSIN) was dissolved in 5 mM phosphate buffer (pH 7.0) with a concentration of 0.1 mg/ml. CD spectra were measured on the Chirascan Plus spectropolarimeter v.4.4.0 (Applied Photophysics Ltd, UK) by using a quartz cell of 1.0-mm thickness. The wavelengths used ranged from 190 to 260 nm. Data were collected at 1-nm intervals with a scan rate of 60 nm/min and expressed as intensity (mdeg).

### Surface plasmon resonance binding experiments

Surface plasmon resonance was used to evaluate the binding of AMSIN, emorisin, and (ΔGFG)AMSIN to various RBDs. WT RBD was prepared according to the method previously described (Gao and Zhu, 2021). Recombinant α, β, γ, ζ RBDs and human ACE2 (hACE2) were purchased from KMD Bioscience (Tianjin, China) (αRBD: Cat No. COV390; βRBD: Cat No. COV380; γRBD: Cat No. COV381; ζRBD: Cat No. COV391; and hACE2: Cat No. KMPH1835). Recombinant δRBD and oSpike were purchased from Sino Biological (Beijing, China) (Cat No. 40592-V08H90). All these purchased protein products were prepared from mammalian cells. The experiments were performed on the Biacore T100 instrument with a CM-5 sensor chip (GE Healthcare Life Sciences, USA) at 25°C according to the method previously described (Zhu et al., 2022). AMSIN and emorisin were covalently linked on the CM5 sensor chip according to the amine coupling strategy (Nikolovska-Coleska, 2015). For immobilization, the CM5 surface was first activated with two injections of 1-ethyl-3-(3-dimethylaminopropyl)-carbodiimide (EDC 0.4 M) and N-hydroxysuccinimide (NHS 0.1 M) (v:v = 1:1) at a flow rate of 10 μl/min, and then, AMSIN or emorisin solubilized in 10 mM sodium acetate, pH 5.5 at a final concentration of 25 μg/Ml, was injected. Non-reacted carboxylic groups on the sensor chip surface were blocked by ethanolamine-HCl (1 M, pH 8.5) for 420 s at a flow rate of 10 μl/min. Using the method described here, WT RBD and hACE2 were, respectively, covalently linked on the CM5 sensor chip at pH 4.5.

For detecting binding, an analyte was 2-fold diluted with the running buffer PBS-T at indicated final concentrations. This buffer contained 0.05% Tween 20 for preventing any non-specific binding in our SPR experiments. Diluted samples were injected at a flow rate of 30 μl/min over the immobilized peptides for 60 s. Dissociation was monitored for 120 s by injecting the running buffer followed by regeneration with 10 mM Glycine (pH 2.5) for 30 s for both AMSIN and emorisin and 1 M NaCl for 60 s for WT RBD at a flow rate of 30 μl/min for the complete removal of specifically and non-specifically bound biological material from the surface. Responses were measured in RUs as the difference between active and reference channels. The binding curve was fitted with the software BIAevaluation v2.0.1 using a 1:1 Langmuir binding model.

To test whether AMSIN could occupy the ACE2-binding surface on RBD, we carried out an ACE2 competitive assay with SPR by evaluating the potential inhibition effect of AMSIN on the binding of WT RBD to the ACE2-CM5 sensor chip. In this assay, AMSIN was set to four different concentrations (10, 2.5, 0.625, and 0 μM) and the RBD to a constant concentration (0.2 μM).

### Evaluation of activation effect of AMSIN and emorisin on RBD binding to ACE2

SARS-CoV-2 Spike-ACE2 Interaction Inhibitor Screening Assay Kit (Item No. 502050, Cayman Chemical, USA) was used to evaluate the possible activation effect of AMSIN and emorisin on the RBD binding to ACE2 according to the manufacturer's instructions.

Although this kit was designed to identify inhibitors of the SARS-CoV-2 Spike and ACE2 interaction, quantification by reading the absorbance originating from horseradish peroxidase (HRP) catalysis makes it also suitable to identify an activator. This assay reflects the amount of the enzyme-linked ACE2 captured by a recombinant rabbit Fc-tagged SARS-CoV-2 Spike S1 RBD that binds to a plate precoated with a mouse anti-rabbit antibody. If the amount is increased, one can infer that the peptide activates the ACE2-binding activity of WT RBD.

### Statistics

Student's *t*-test was used to compare the means between the two groups with SPSS Statistics 17.0 (SPSS Inc.).

## Results

### Emorisin shares a conserved structural motif with AMSIN and adopts a defensin fold

**Figure 1:**
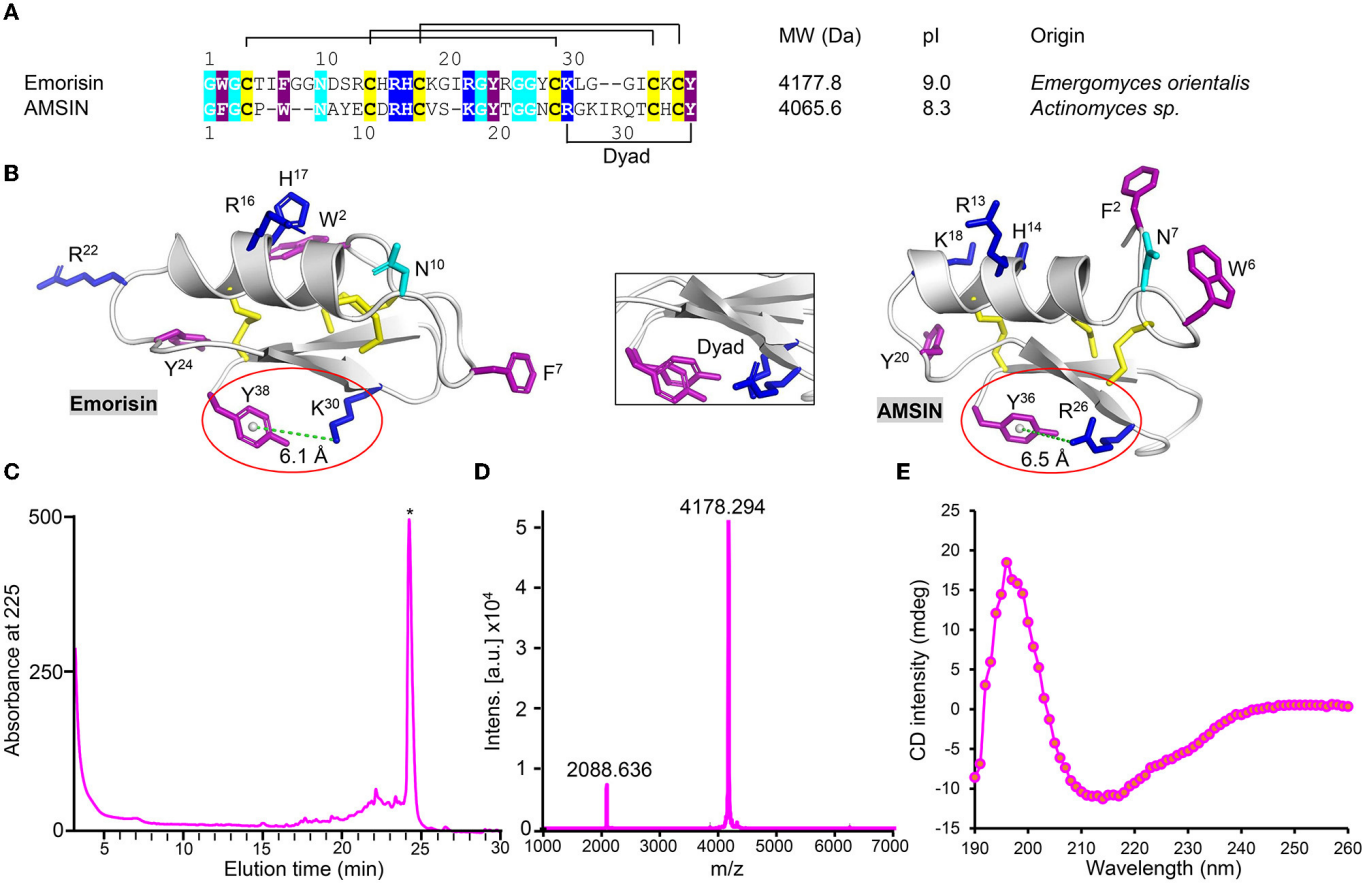
Identification of emorisin. **(A)** Sequence comparison between AMSIN and emorisin. Conserved residues are shaded in color, with polar in cyan, cationic in blue, aromatic in purple, and cysteines in yellow. **(B)** Mapping of conserved residues on structures of AMSIN and emorisin. The residues are displayed as sticks with the same color codes as those in **(A)**. The structurally conserved dyad motif comprising the aromatic Tyr-38 in emorisin or Tyr-36 in AMSIN and the cationic Lys-30 in emorisin or Arg-26 in AMSIN are circled in red. The distance between these residues (6.1 or 6.5Å) is indicated by a green dotted line. Inset: structural superimposition of the dyad between AMSIN and emorisin. **(C)** Purification of the air-oxidized emorisin (denoted by the asterisk) by RP-HPLC. The Agilent Zorbax 300SB-C18 (4.6 × 150mm, 5μm) was equilibrated with 0.05% TFA in water (v/v), and peptides were eluted from the column with a linear gradient from 0 to 60% acetonitrile in 0.05% TFA within 40 min with a flow rate of 1 ml/min. The UV absorbance was monitored spectrophotometrically at 225 nm. **(D)** MALDI-TOF. The two main peaks, respectively, correspond to the singly and doubly protonated forms. **(E)** CD spectroscopy analysis of emorisin.

Using the gene discovery strategy, we found a new fungal defensin from the human pathogenic fungus *Emergomyces orientalis* (GenBank No. MOWL01000070.1) (He et al., 2021), named emorisin. This peptide is composed of 38 amino acids with three predicted disulfide bridges (Figure 1A). Compared with the functionally known defensins, emorisin shares the highest sequence similarity (57.5%) to plectasin, the prototype of fungal defensins (Mygind et al., 2005). Using a structural modeling technique (for the templates used, see Supplementary Figure S1), we identified a structural motif in emorisin, which is comprised of the cationic residue Lys-30 and the aromatic residue Tyr-38, both having a distance of 6.1Å when calculated from the center of the aromatic ring to the Lys NZ atom (Figure 1B). Such a motif has been named dyad previously in some structurally unrelated K^+^ channel toxins from various animals, such as scorpions, cone snails, snakes, and sea anemones, in which it is involved in interaction with K^+^ channels (Dauplais et al., 1997; Gao et al., 2013). Intriguingly, this motif also exists in AMSIN (Figure 1B) but lacks in plectasin (Supplementary Figure S1). Since AMSIN and emorisin share only 40% sequence similarity, the conservation of this dyad could be of certain functional significance.

To study its structure and function, we prepared native-like emorisin by air oxidization of its chemically synthesized reduced form in an alkaline environment. The refolded product was purified to homogeneity by RP-HPLC, where it was eluted at a retention time of 24 min (Figure 1C). Using MALDI-TOF, we verified its experimental molecular weight (MW) (4,178.294 Da), which perfectly matched its theoretical MW (4,177.8 Da) calculated from its oxidized form (Figure 1D). CD spectroscopy analysis of the folded emorisin revealed a minimum at 212 nm and a maximum at 196 nm (Figure 1E), demonstrating that it has adopted a native CSαβ-type defensin conformation (Zhu et al., 2012). To understand the structure–function relationship of AMSIN, we produced a chemically synthesized mutant [herein named (ΔGFG)AMSIN] with its three N-terminal residues (Gly–Phe–Gly) deleted (Supplementary Figures S2A, B). CD analysis showed that this deletion did not alter the structure of AMSIN, as identified by the refolded mutant having similar CD signatures at the minimum and maximum absorbance to the unmodified peptide (Supplementary Figure S2C).

### AMSIN and emorisin are two novel binders of SARS-CoV-2 RBDs

**Figure 2:**
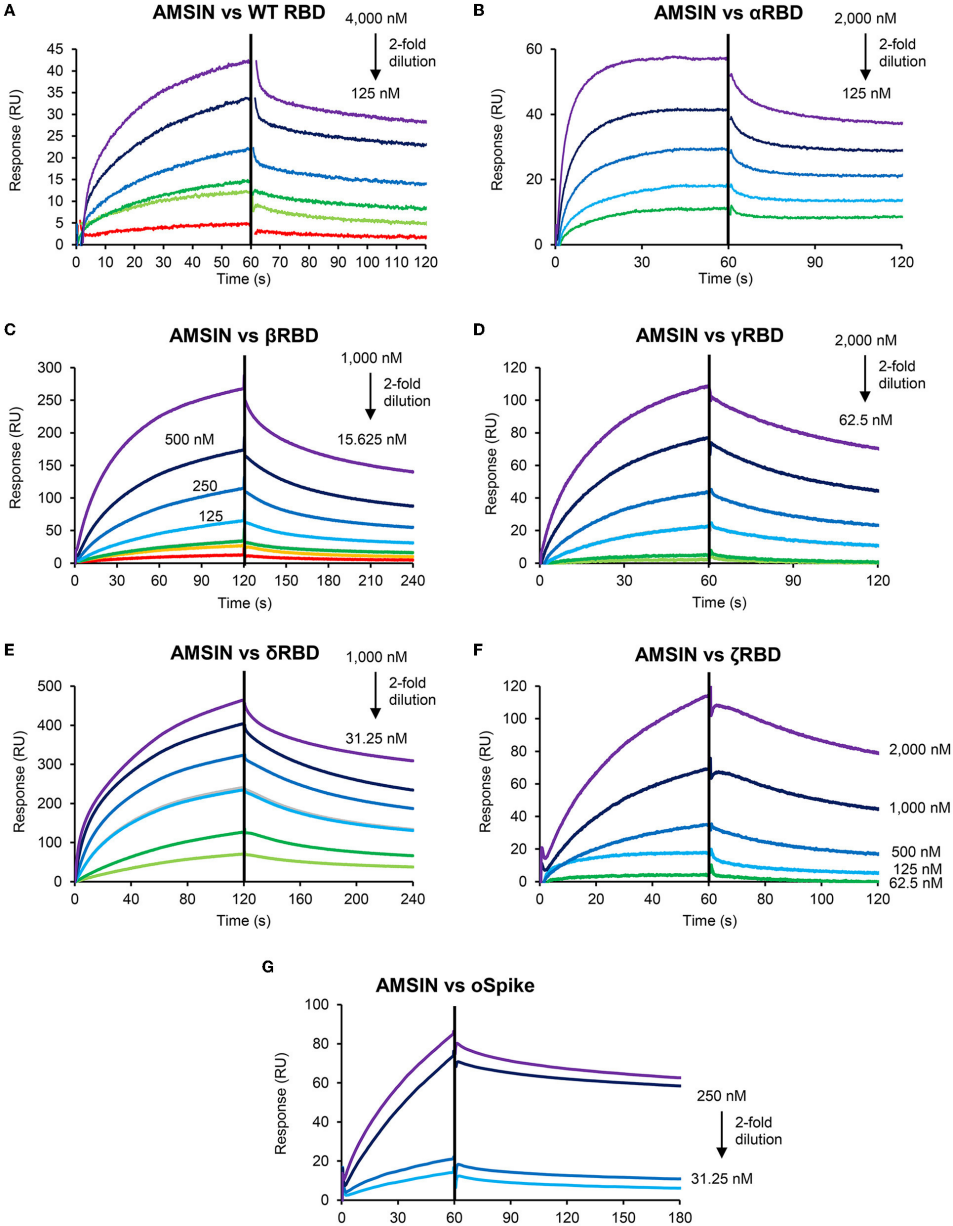
Sensorgrams of SARS-CoV-2 RBD binding to the AMSIN-immobilized chip surface. The concentrations with 2-fold serial dilutions are shown here. In this SPR experiment, AMSIN as the ligand was covalently immobilized onto CM5 via its amine groups, and the analytes (various RBDs) flowed over the surface. **(A)** WT RBD; **(B)** αRBD; **(C)** βRBD; **(D)** γRBD; **(E)** δRBD; **(F)** ζRBD; and **(G)** oSpike.

**Figure 3:**
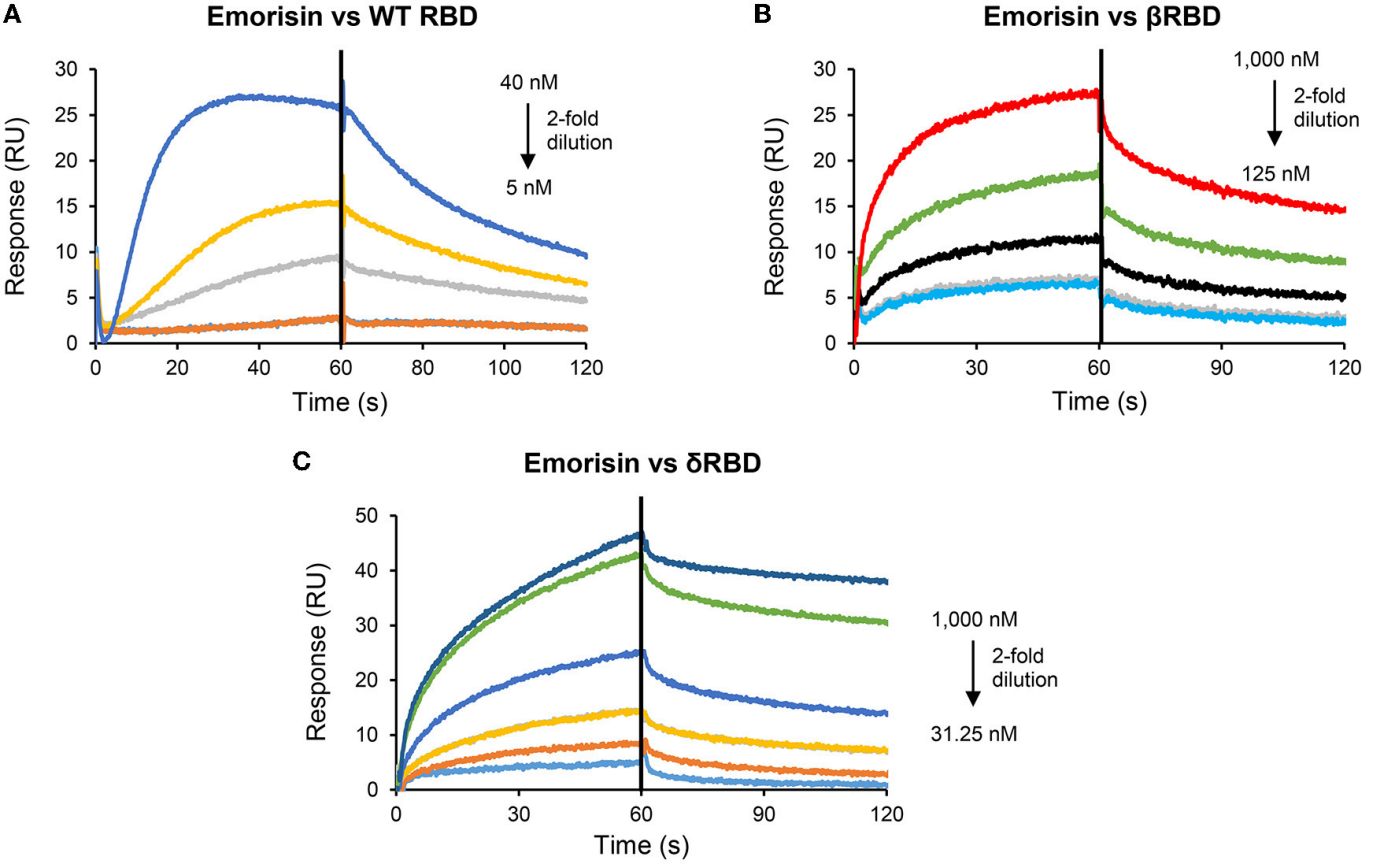
Sensorgrams of SARS-CoV-2 RBD binding to the emorisin-immobilized chip surface. The concentrations with 2-fold serial dilutions are shown here. In this SPR experiment, emorisin as the ligand was covalently immobilized onto CM5 via its amine groups, and the analytes (various RBDs) flowed over the surface. **(A)** WT RBD; **(B)** βRBD; and **(C)** δRBD.

Using SPR experiments, we studied the interactions between the two peptides and SARS-CoV-2 Spike RBD proteins, in which the peptides were covalently immobilized onto CM5 chips and the analytes (various RBDs) flowed over the surface. RBD proteins used were derived from WT and various variants (α, β, γ, δ, and ζ) (Table 1). The results showed that AMSIN exhibited a high affinity to αRBD and δRBD with a K_D_ of 37.4 and 76.6 nM, respectively, and a moderate affinity to other RBDs with a K_D_ ranging from 193 to 1,450 nM. The association constant (K_on_) of AMSIN over all the RBDs ranged between 3.46 × 10^3^ and 4.96 × 10^4^ M^−1^s^−1^ and the dissociation constant (K_off_) between 1.16 × 10^−3^ and 5.45 × 10^−3^ s^−1^ (Figure 2; Table 1). Emorisin exhibited a high affinity to WT RBD, βRBD, and δRBD with a K_D_ of 7.6, 49.5, and 31.6 nM, respectively, and the association constant (K_on_) ranged between 1.14 × 10^4^ and 5.92 × 10^6^ M^−1^s^−1^ and the dissociation constant (K_off_) between 4.50 × 10^−2^ and 5.61 × 10^−3^ s^−1^ (Figure 3; Table 1). In addition, SPR measurements showed that the refolded (ΔGFG)AMSIN bound to WT RBD with a K_D_ of 119 nM (Supplementary Figure S2D), roughly equivalent to that of AMSIN (Table 1), indicating that the three N-terminal residues are not involved in interaction with WT RBD. Given that the Omicron variant is currently dominant, we also measured the binding of AMSIN to its Spike protein. The result showed that AMSIN was capable of binding the protein with a high affinity (K_D_ of 57.9 nM). The association (K_on_) and dissociation (K_off_) constants were 3.17 × 10^4^ M^−1^s^−1^ and 1.83 × 10^−3^ s^−1^, respectively (Figure 2; Table 1).

**Table 1:** Affinity and kinetics of AMSIN and emorisin on various RBDs from SARS-CoV-2 and its variants.

	**AMSIN**			**Emorisin**		
	**K**_D_ **(nM)**	**K**_on_ **(M**^−1^**s**^−1^**)**	**K**_off_ **(s**^−1^**)**	**K**_D_ **(nM)**	**K**_on_ **(M**^−1^**s**^−1^**)**	**K**_off_ **(s**^−1^**)**
WT RBD^*^	193	1.13 × 10^4^	2.19 × 10^−3^	7.6	5.92 × 10^6^	4.50 × 10^−2^
αRBD^**^	37.4	3.09 × 10^4^	1.16 × 10^−3^	n.d.	n.d.	n.d.
βRBD^**^	310	1.73 × 10^4^	5.36 × 10^−3^	49.5	1.14 × 10^4^	5.61 × 10^−3^
γRBD^**^	607	8.97 × 10^3^	5.45 × 10^−3^	n.d.	n.d.	n.d.
δRBD^**^	76.6	4.96 × 10^4^	3.80 × 10^−3^	31.6	6.40 × 10^4^	2.02 × 10^−3^
ζRBD^**^	1,450	3.46 × 10^3^	5.02 × 10^−3^	n.d.	n.d.	n.d.
oSpike^**^	57.9	3.17 × 10^4^	1.83 × 10^−3^	n.d.	n.d.	n.d.

^*^Prepared from *E. coli*; ^**^ prepared from mammalian cells. “n.d.” means “not determined.”

### AMSIN and emorisin both activate the ACE2-binding activity of RBD

**Figure 4:**
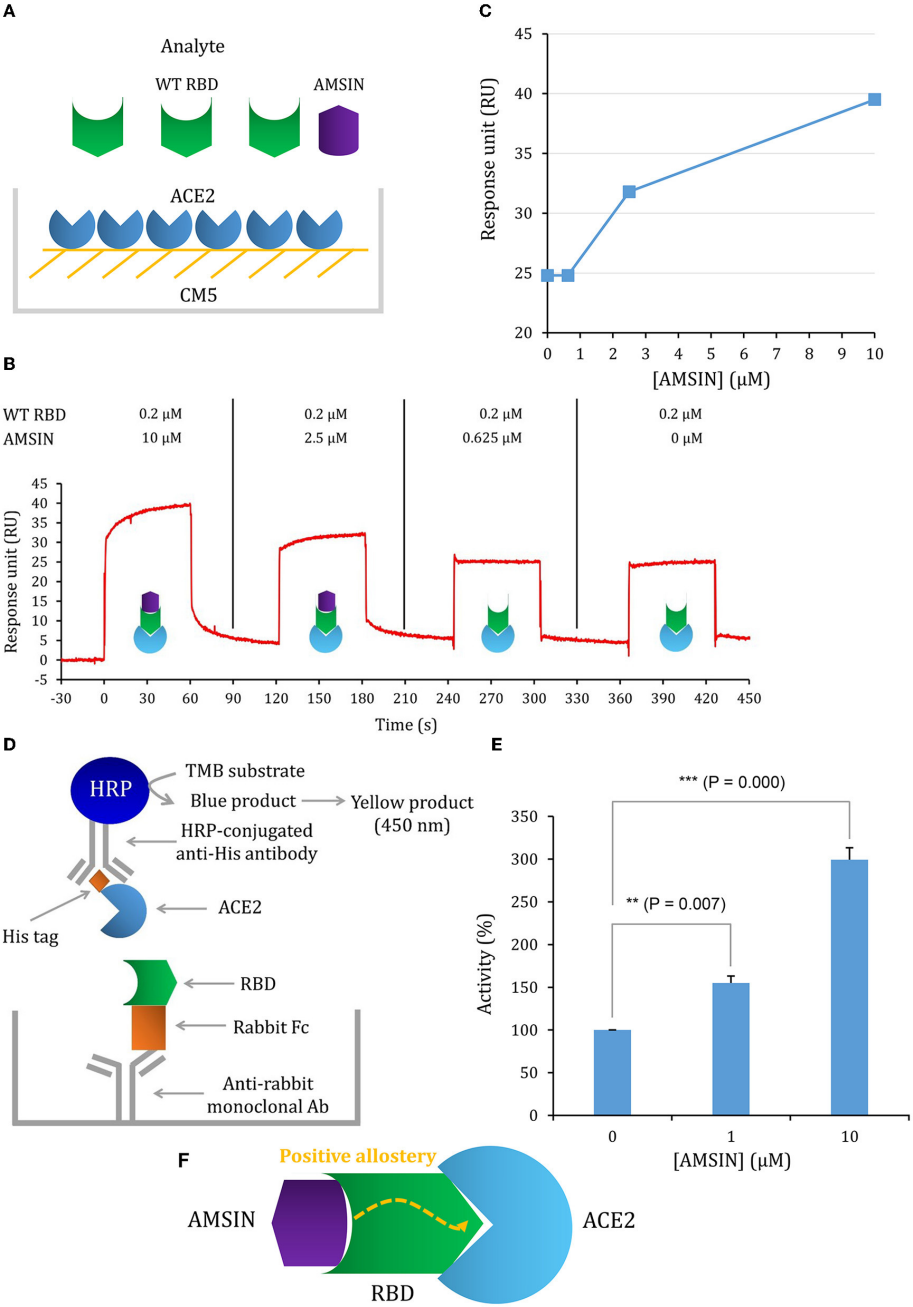
AMSIN is an activator of RBD binding to ACE2. **(A)** Schematic diagram of competitive SPR experiment, in which the ligand hACE2 was covalently immobilized onto CM5 via its amine groups and the analyte—the WT RBD flowed over the surface, with AMSIN was added at different concentrations; **(B)** The sensorgrams of the ACE2 competitive binding assay. In this assay, WT RBD was set to a constant concentration (200 nM) and AMSIN to different concentrations as indicated here. The possible surface complexes formed are also shown; **(C)** The comparisons of RU values recorded in the presence of different concentrations of AMSIN; **(D)** A schematic diagram of the SARS-CoV-2 Spike-ACE2 Interaction Inhibitor Screening Assay Kit for assaying the effect of AMSIN on the binding of WT RBD to ACE2. A recombinant His-tagged ACE2 protein binds the Spike RBD that binds to a plate precoated with a mouse anti-rabbit antibody, and the complex is detected with an HRP-conjugated anti-His antibody, which is easily quantified by reading the absorbance at 450 nm. A control is included for the competition of the SARS-CoV-2 Spike RBD-ACE2 interaction; **(E)** AMSIN activates the effect of WT RBD binding to ACE2. *P*-values were obtained by Student's *t*-test: **P < 0.01 and ***P < 0.001; **(F)** A schematic drawing of positive allostery elicited by AMSIN binding to WT RBD, which enhances the binding of the latter to ACE2.

**Figure 5:**
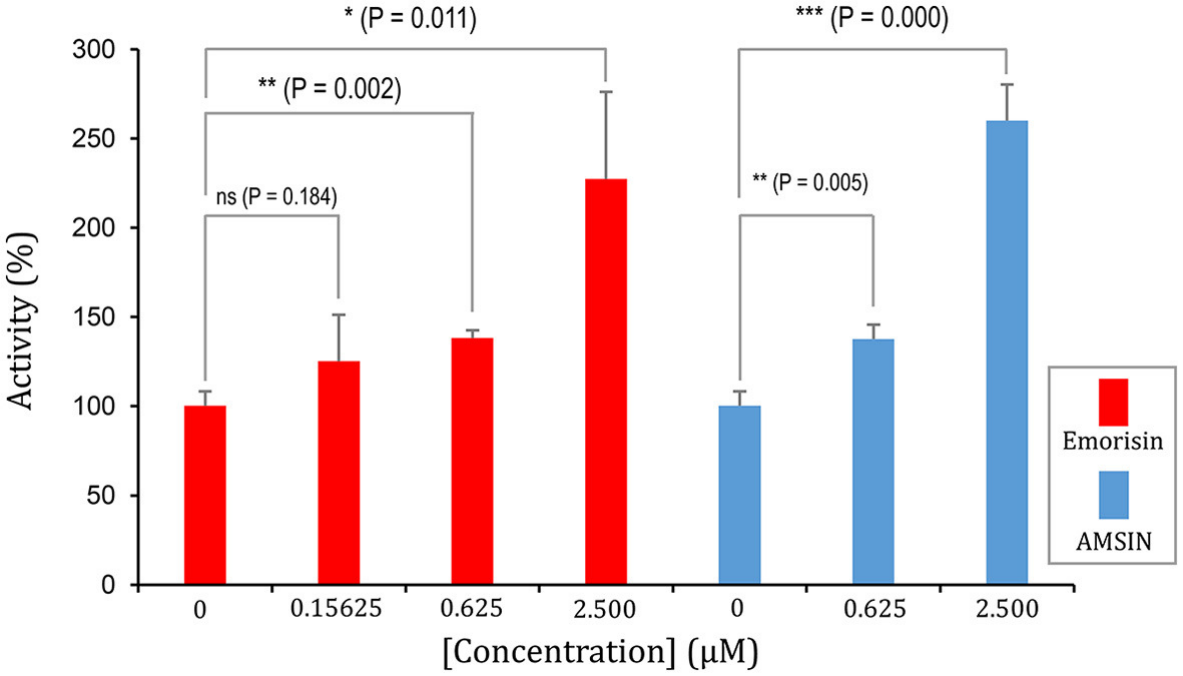
Emorisin activates the effect of WT RBD binding to ACE2. *P*-values were obtained by Student's *t*-test: *P < 0.05, **P < 0.01, and ***P < 0.001, ns, no significance. AMSIN was measured in parallel.

To study whether these two peptides occupy a similar surface with ACE2 on RBDs, we conducted the ACE2 competitive binding experiment using SPR. It was shown that when AMSIN (0, 0.625, 2.5, or 10 μM) was added to the analyte (0.2 μM WT RBD) to flow over the ACE2-CM5 chip surface, response units (RUs) originated from the binding of the RBD to ACE2 increased and this response occurred in a concentration-dependent manner (Figures 4A–C), indicating that AMSIN did not interfere with the RBD-ACE2 binding and shared no common interacting surface with ACE2. Interestingly, it seemed that AMSIN could activate the ACE2-binding function of WT RBD. In order to provide further evidence, we employed an ELISA method to monitor the binding increase in the presence of the peptide. In this ELISA kit, the RBD was bound to a plate, and ACE2 was linked to an HRP enzyme. This experiment can indirectly measure the amount of change of the ACE2-RBD complex by the enzyme catalysis of its substrate and quantified by reading the absorbance at 450 nm (Figure 4D). We found that in line with the SPR result, AMSIN was able to significantly enhance the binding of WT RBD to ACE2 (*P* < 0.01) in a concentration-dependent manner (Figure 4E). Taken together, these two different methods yielded consistent results, and thus, our studies have identified AMSIN as a modulator of WT RBD that enhance the ACE2-binding activity likely via a positive allostery mechanism (Figure 4F). Using the same method, we showed that emorisin had a similar functional feature (Figure 5).

### Possible structural underpinnings of AMSIN-evoked allostery in RBD

**Figure 6:**
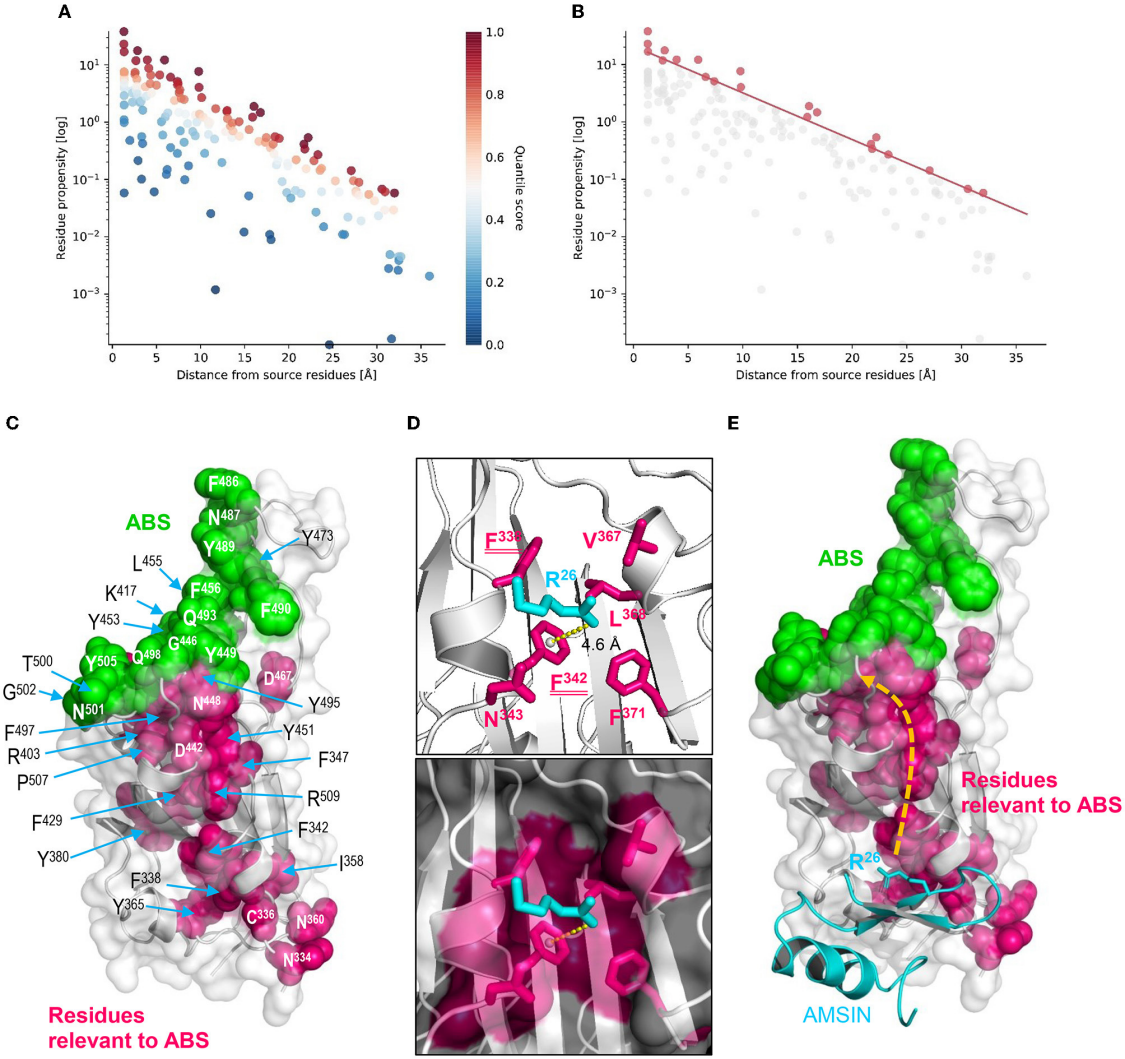
Structural underpinnings of AMSIN-evoked allostery in RBD. The residue relevance was predicted by ProteinLens. **(A)** The hotspot view of high or low connectivity to the ABR as source sites. All data points were plotted as propensity over distance from the source, in which hotspots and coldspots are colored according to their quantile scores; **(B)** The relevant residue view at 0.90 quantile score cutoff; **(C)** Mapping of the RBD residues (*pink*) relevant to its ABSs (*green*) on the structure of the WT RBD (PDB entry 6LZG); **(D)** Arg-26 of AMSIN interacting with residues of the RBD, in which two belong to those relevant to the ABSs (namely Phe-338 and Phe-342), underlined twice here. A cation-π interaction exists between Arg-26 and Phe-342, shown by a *yellow* dotted line; **(E)** A proposed allosteric pathway in the RBD driven by the binding of Arg-26 of AMSIN, leading to the enhancement of ACE2 binding in WT RBD.

In order to explore the possible structural underpinnings of the peptide-evoked allostery in RBD, we first analyzed the residues of WT RBD relevant to its ABSs using ProteinLens (Figures 6A, B), from which a total of 21 residues are identified (Figure 6C). These residues structurally cluster together and physically connect to the RBD ABSs (Figure 6C). This suggests that this cluster of relevant residues may serve as a “wire” to transfer the peptide binding signal to the ABSs via allostery to enhance ACE2 binding. In addition to WT RBD, we found that various variant RBDs, such as α, β, γ, δ, and o, also possess an overall similar relevant residue distribution relative to their ABSs (Supplementary Tables S1, S2; Supplementary Figures S3–S5), in which nine sites (Phe-338, Phe-342, Phe-347, Phe-429, Asp-442, Tyr-451, Phe-497, Pro-507, and Arg-509) are completely identical, suggesting the conservation of allosteric communication in these RBDs. Intriguingly, a deep mutational scanning of SARS-CoV-2 RBD revealed that most saturated mutations at these sites lowered the binding affinity to ACE2 (Starr et al., 2020), highlighting their functional importance. As the locations of these residues are remote from the RBD-ACE2 interface, such an effect could be achieved through allosteric communication with the ABSs. In addition, Phe-497 has also been identified as critical for the RBD-ACE2 interaction based on computational saturation mutagenesis approaches (Teng et al., 2021).

To study whether these relevant residues mediate the AMSIN-elicited allostery in RBD, we created an AMSIN-RBD complex by molecular docking and chose a plausible complex model through filtering with the following four criteria: (1) The amino-terminal triplet motif “Gly–Phe–Gly” of AMSIN was excluded in the interactions; (2) the ACE2-binding face of RBD was excluded; (3) the region around glycosylated Asn-343 of RBD was excluded; and (4) the conserved dyad motif in AMSIN was considered in the interactions. Based on these criteria, we carried out two rounds of filtering and chose the most plausible model for further analysis (Supplementary Figure S6). The model predicts a key interaction network formed between Arg-26, one of the dyads in AMSIN, and six RBD residues, namely Phe-338, Phe-342, Asn-343, Val-367, Leu-368, and Phe-371 (Figure 6D), in which two aromatic residues (Phe-338 and Phe-342) belong to relevant residues of ABSs. In particular, the CaPTURE program identified a cation-π interaction on the AMSIN-WT RBD interface, which was contributed by Arg-26 of AMSIN and Phe-342 of WT RBD with a distance of 4.6Å (Supplementary Table S3; Figure 6D). In addition, Tyr-36 of AMSIN, another dyad residue of AMSIN, was found to interact with Asn-343 of WT RBD in the complex model. We thereby proposed that this cation-π interaction could primarily trigger the allosteric communication in the RBD via transferring the ligand-binding signal to the distant ABSs to enhance the ACE2-binding activity via a conformational adjustment in their ABSs (Figure 6E). Although this proposal is in line with the classical theory of allostery (namely, proteins propagate the effect of ligand binding at the allosteric site to a spatially distant orthosteric site to affect activity), its feasibility needs more experimental data to support, in which the determination of the interacting distance between Arg-26 of AMSIN and Phe-342 of WT RBD using the double mutant cycle methodology (Pagano et al., 2021) and of the complex structure of the peptide, the RBD and ACE2 with the cryo-electron microscopy technique (Danev et al., 2019) could be the first step in understanding the role of peptide binding as an allosteric trigger (Gianni and Jemth, 2023).

### AMSIN-CM5 chip could be used as a diagnostic agent for RBD detection

**Figure 7:**
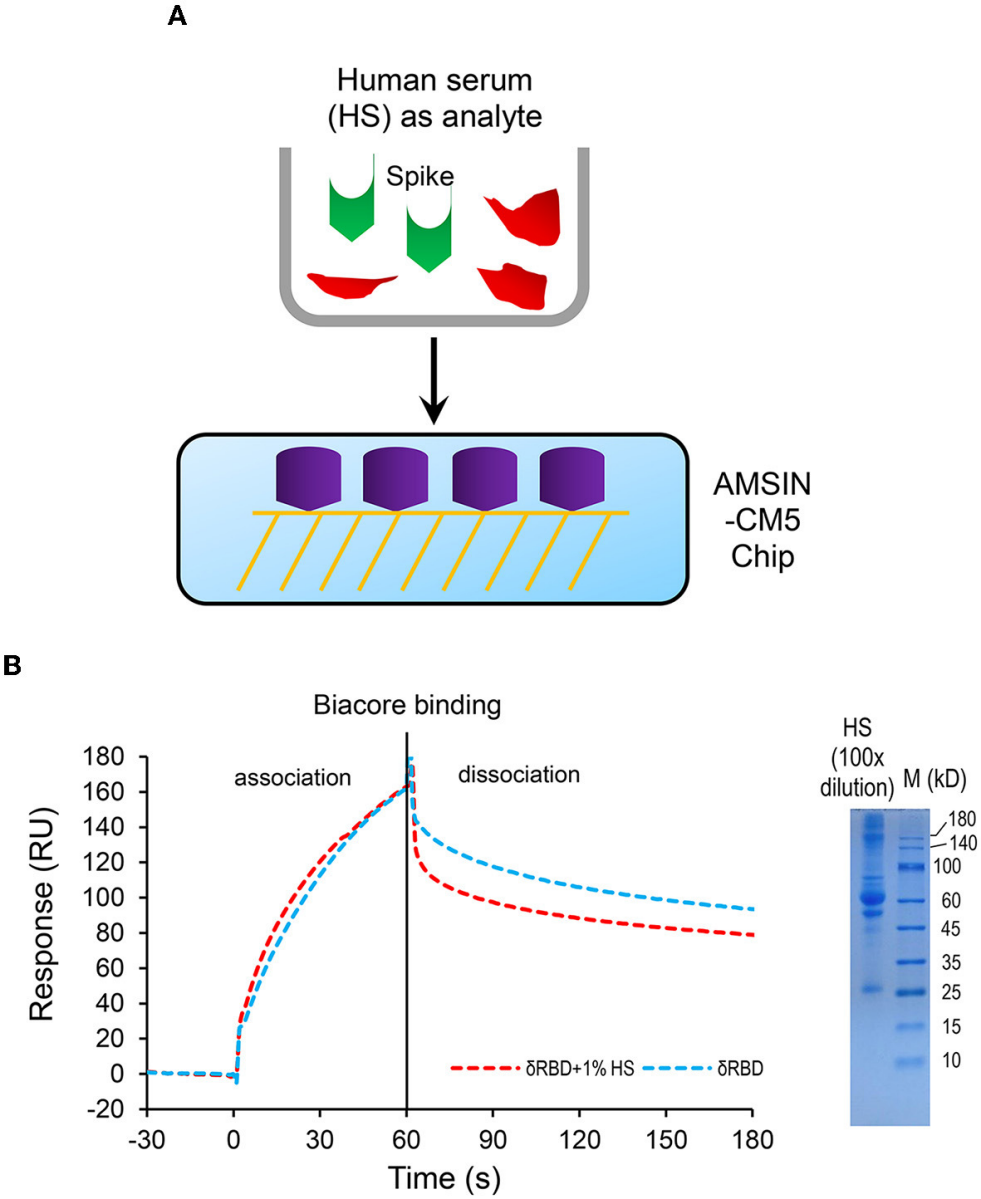
The human serum does not interfere with the binding of δRBD to AMSIN covalently immobilized onto CM5. **(A)** A schematic diagram of competitive SPR experiment; **(B)** Sensorgrams of δRBD (250 nM) binding to the AMSIN-immobilized chip surface in the presence or absence of 1% human serum. Right: SDS-PAGE showing the protein components of 1% human serum. The concentration of δRBD used was 250 nM.

In this study, we found that AMSIN was rather stable when covalently immobilized onto CM5 via their amine groups. This feature probably makes it a useful tool for detecting SARS-CoV-2 in human serum. To test this probability, we compared its binding to δRBD as a representative in the absence and presence of human serum with SPR. The result showed that 1% human serum did not interfere with the binding (Figure 7), highlighting the potential of the AMSIN-CM5 chip as a diagnostic agent for RBD detection.

## Discussion

In this study, we identified two new RBD binders. Since AMSIN can bind WT RBD and a series of variant RBDs, it is reasonable to infer that this peptide interacts with a conserved region of these RBDs. This is highly consistent with our computational model (Figure 6), in which 12 WT RBD residues are identified to directly interact with AMSIN (Supplementary Figure S7). All these residues are completely conserved in α, β, γ, δ, and ζ RBDs and mostly conserved in Orbd, and they all are located on the N-terminus (Supplementary Figure S7). The affinity differential among RBDs (Table 1) suggests that in addition to the 12 residues, others could act as secondary contacts involved in interaction with AMSIN.

Mutations in RBDs far away from the RBD-ACE2 interface have been proposed to play a role in improving their ACE2-binding capacity via allosteric communication. For example, in the SARS-CoV-2 delta variant RBD, two single mutations (E484Q or T478K) were found to impact the RBD-ACE2 binding in a distinct manner (Chan et al., 2022). Mutation-induced long-range allosteric interactions in the Spike protein were also found to determine the infectivity of SARS-CoV-2, in which each RBD mutation acts like a positive allosteric modulator (Das et al., 2021). In addition, a network-centric adaptation model of the reversed allosteric communication has established a robust connection between allosteric network hotspots and potential allosteric binding pockets in the Omicron RBD-ACE2 complexes (Verkhivker et al., 2022). All these observations suggest that mutations can elicit allostery in RBDs. Mutations at some non-RBD regions, e.g., the cleavage site of the SARS-CoV-2 Spike protein, were also found to influence protein stability and cell–cell fusion, suggesting the presence of a wider allosteric effect in this protein (Barrett et al., 2021). In addition to mutations, ligand binding is also a perturbation factor evoking allostery. For example, in hemoglobin (Hb), ligand binding has far more ramifications, not just on the heme iron, but rather on the structure of Hb by inducing conformational changes to give rise to allostery (Ahmed et al., 2020). In G protein-coupled receptors (GPCRs), ligand binding and G protein interaction are achieved via allosteric coupling (Weis and Kobilka, 2018). Using molecular dynamic simulations, Bhattacharjee et al. observed the allosteric crosstalk within the RBD in the *apo*- and the ACE2 receptor-bound states (Bhattacharjee et al., 2021). Our studies showed that the two microbial defensins were capable of regulating the ACE2-binding activity of RBDs in an allosteric manner likely via a cation-π interaction as the trigger of allostery. The role of the cation-π interaction in allostery has been previously observed in other proteins. For example, a structurally conserved cation-π lock between transmembrane helix VI (TM6) and H8 (TM7) regulates cytoplasmic cavity opening as a “gatekeeper” for G protein penetration via allostery (Wang et al., 2023). Using mutational analyses, Chinnaraj et al. (2022) demonstrated that the R300-W396 cation-π interaction dictates how the catalytic domains of human protein disulfide isomerase (PDI) relocate.

These two microbial defensins could have potential values in the following aspects: (1) as a research tool for investigating their impacts on viral infectivity at the cellular and organismal levels; (2) once the peptide-RBD complex structure is experimentally established, they could be useful as an inhibitor of RBD-ACE2 interaction with some therapeutic value when fused with other molecules. In addition, the recognition of the RBD residues relevant to ACE2 binding could be valuable in the prediction of the effects of the mutations at these sites on the viral infection; (3) as a biotechnological tool for affinity purification of Spike proteins of interest. In addition to these two peptides, a database survey revealed that there are other defensins from bacteria and ticks also containing a dyad (Supplementary Figure S8) that need to be identified in the future for their activating effect on SARS-CoV-2 RBDs.

Previous studies have shown that several human-sourced α-type (e.g., HNP1–HNP3), β-type (e.g., HBD-2), and θ-type (i.e., RC-101) defensins can inhibit SARS-CoV-2 infection by blocking viral entry either through inhibiting viral fusion (Xu et al., 2021) or the interaction of Spike with the ACE2 receptor (Kudryashova et al., 2022; Zhang et al., 2022). In addition, a fungal defensin was reported to possess the ACE2-binding ability, which could occupy the ACE2-binding region in RBD (Gao and Zhu, 2021). Different from these peptides, AMSIN and emorisin both do not competitively bind with ACE2 but bind sites far away from the ABS to exert a role via allostery. This uniqueness makes them valuable tools for investigating the mutational effect of SARS-CoV-2 RBDs in an allosteric manner.

In summary, this study identifies for the first time two new binders of SARS-CoV-2 RBD proteins with a positive allosteric effect on their binding to ACE2. A cation-π interaction was proposed to act as a trigger for driving conformational transduction in the RBDs, in which the ligand-binding signal is transferred to their interface to elicit an enhanced ACE2 binding. Further determination of the complex structure of these peptides and RBDs will validate our proposal and expand the application values of these peptides either as research tools or specific targeting peptides for drug leads when a fusion protein could be designed based on the structure information to impair the binding of Spike to ACE2 via forming steric hindrance.

## Data availability statement

The raw data supporting the conclusions of this article will be made available by the authors, without undue reservation.

## Author contributions

SZ conceived and designed this study and performed sequence and structural analyses. BG performed experiments. BG and SZ commonly wrote the study. Both authors contributed to the article and approved the submitted version.
