# Supplementary material for: Enhancement of SARS-CoV-2 receptor-binding domain activity by two microbial defensins
Source: Front Microbiol. 2023 Jun 19;14:1195156. doi: 10.3389/fmicb.2023.1195156 (PMC10315472; doi:10.3389/fmicb.2023.1195156)
Supplement: Supplementary file 1 [file Data_Sheet_1.PDF]

Supplementary Material

Table S1. Functional residues of RBDs involved in interacting with ACE2

	417	446	449	453	455	456	473	475	476	477	486	487	489	490	493	494	496	498	500	501	502	503	505
WT	K	G	Y	Y	L	F	Y	A	G	-	F	N	Y	F	Q	-	G	Q	T	N	G	-	Y
α	-	G	Y	Y	L	F	-	A	G	S	F	N	Y	-	Q	-	-	-	T	Y	G	-	Y
β	-	G	Y	Y	L	F	-	A	G	-	F	N	Y	-	Q	-	-	Q	T	Y	G	V	Y
γ	-	G	Y	Y	L	F	-	A	G	-	F	N	Y	-	Q	-	-	Q	T	Y	G	-	Y
δ	K	G	Y	Y	L	F	Y	A	-	-	F	N	Y	-	Q	-	G	Q	T	N	G	-	Y
o	-	-	Y	Y	-	F	Y	A	G	-	F	N	Y	-	R	S	S	R	T	Y	G	-	H

Note: Conserved residues involved in ACE2 binding among variants are colored red, which were used as source residues for ProteinLens analysis. PDB entries for the structures used here are: 7FEM (αRBD); 7EKGβ (RBD); 7EKC (γRBD); 7WBQ (δRBD); and 7WBP (oRBD).

Table S2. Predicted residues relevant to ACE2 binding in various RBDs by ProteinLens

	334	336	338	342	347	356	358	360	365	366	369	374	380	382	386	388	392	400	401	403	406	410	429	430	438	442	448	451	464	467	469	495	497	507	509	510	515	523
WT	N	C	F	F	F	-	I	N	Y	-	-	-	Y	-	-	-	-	F	-	R	-	-	F	-	-	D	N	Y	-	D	-	Y	F	P	R	-	F	-
α	-	-	F	F	F	K	I	-	-	-	-	F	-	V	-	-	F	-	-	-	-	I	F	T	S	D	-	Y	F	-	-	Y	F	P	R	V	F	-
β	N	-	F	F	F	-	-	N	Y	-	-	-	Y	-	-	-	F	F	V	-	-	-	F	-	S	D	-	Y	F	-	-	Y	F	P	R	-	F	-
γ	N	-	F	F	F	-	-	N	Y	S	Y	-	Y	-	-	-	-	F	V	-	E	-	F	-	S	D	-	Y	F	-	-	Y	F	P	R	-	F	-
δ	-	-	F	F	F	-	-	N	Y	S	-	-	-	-	K	N	-	F	V	-	-	-	F	-	-	D	N	Y	-	D	-	-	F	P	R	-	-	-
o	N	-	F	F	F	-	-	N	Y	-	-	-	Y	-	-	N	-	-	-	-	-	-	F	-	S	D	-	Y	F	-	S	Y	F	P	R	-	-	T

Note: Sites involved in direct interaction with Arg-26 of AMSIN are shaded in grey. Conserved residues relevant to ACE2-binding sites are colored in red.

Table S3. Cation-π prediction in the interface of the computational model of AMSIN-WT RBD by CAPTURE

Cation	AA #	Chain	Pi	AA #	Chain	E(es) (kcal/mol)	E(vdw) (kcal/mol)
Arg	26	A (AMSIN)	Phe	342	B (WT RBD)	-1.92	-1.89

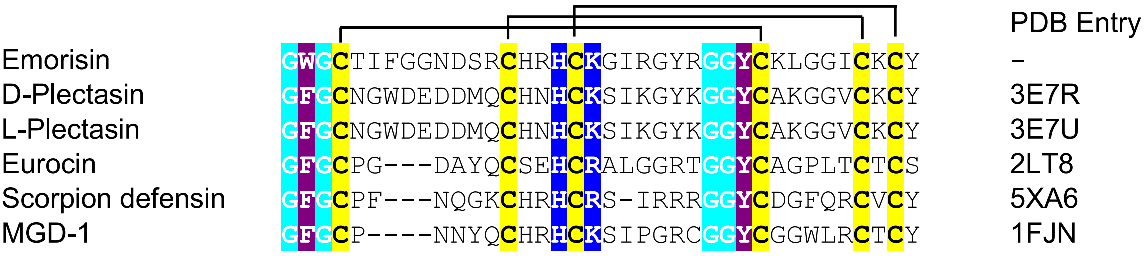

Figure S1. Homologous templates recognized by trRosetta (https://yanglab.nankai.edu.cn/trRosetta/) for building the model of emorisin. In the alignment, identical residues to emorsin are shaded in color, with polar in *cyan*, cationic in *blue*, aromatic in *purple* and Cys in *yellow*.

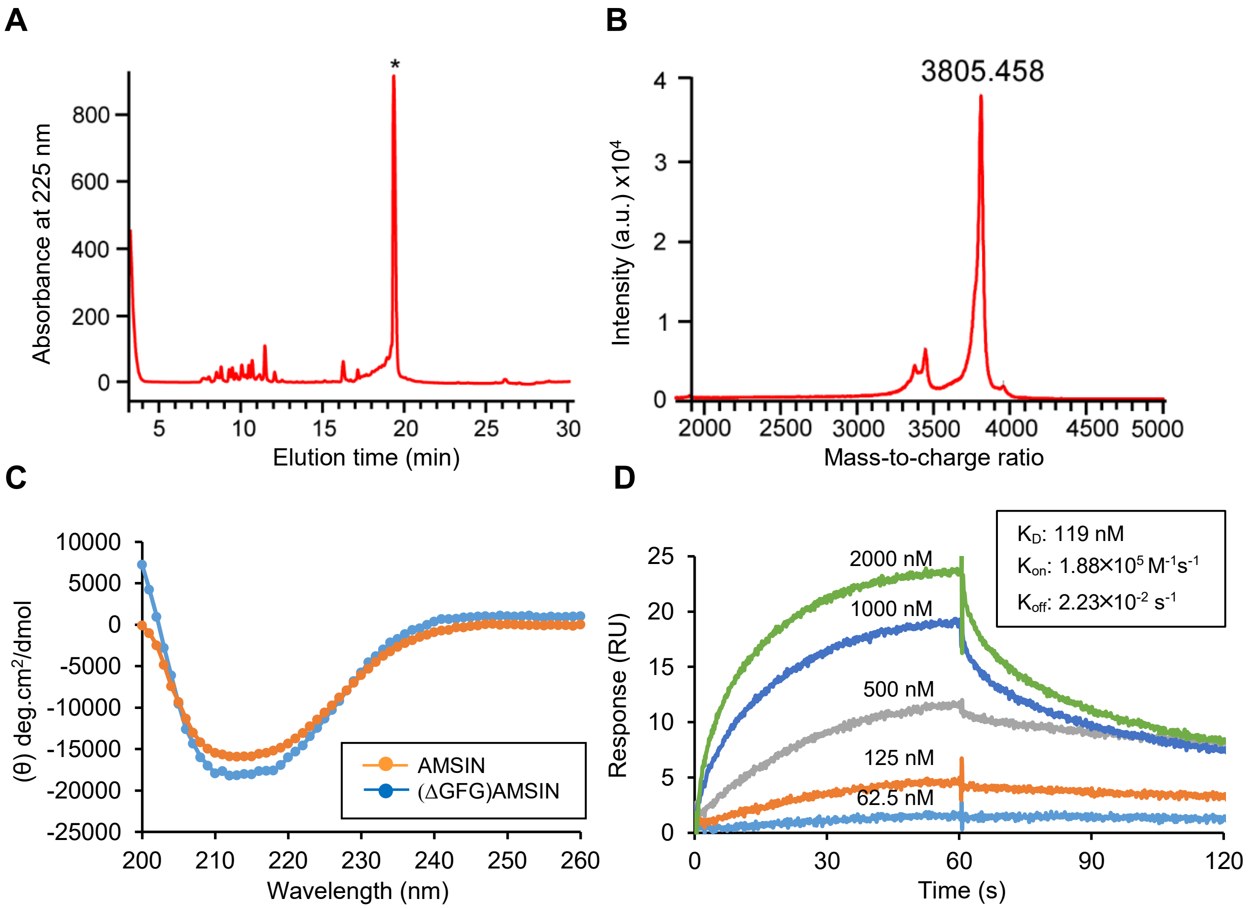

Figure S2. Oxidative refolding and characterization of (ΔGFG)AMSIN. (***A***) Purification of the refolded (ΔGFG)AMSIN by RP-HPLC. C_{18} column was equilibrated with 0.05% TFA and peptides were eluted from the column with a linear gradient from 0% to 60% acetonitrile in 0.05% TFA within 40 min. "*" denotes the correct product; (***B***) MALDI-TOF of the purified peptide; (***C***) Comparison of CD spectra of (ΔGFG)AMSIN and AMSIN. (***D***) Determination of affinity and kinetics of (ΔGFG)AMSIN vs WT RBD with SPR. In this assay, WT RBD was used as the ligand and (ΔGFG)AMSIN as the analyte.

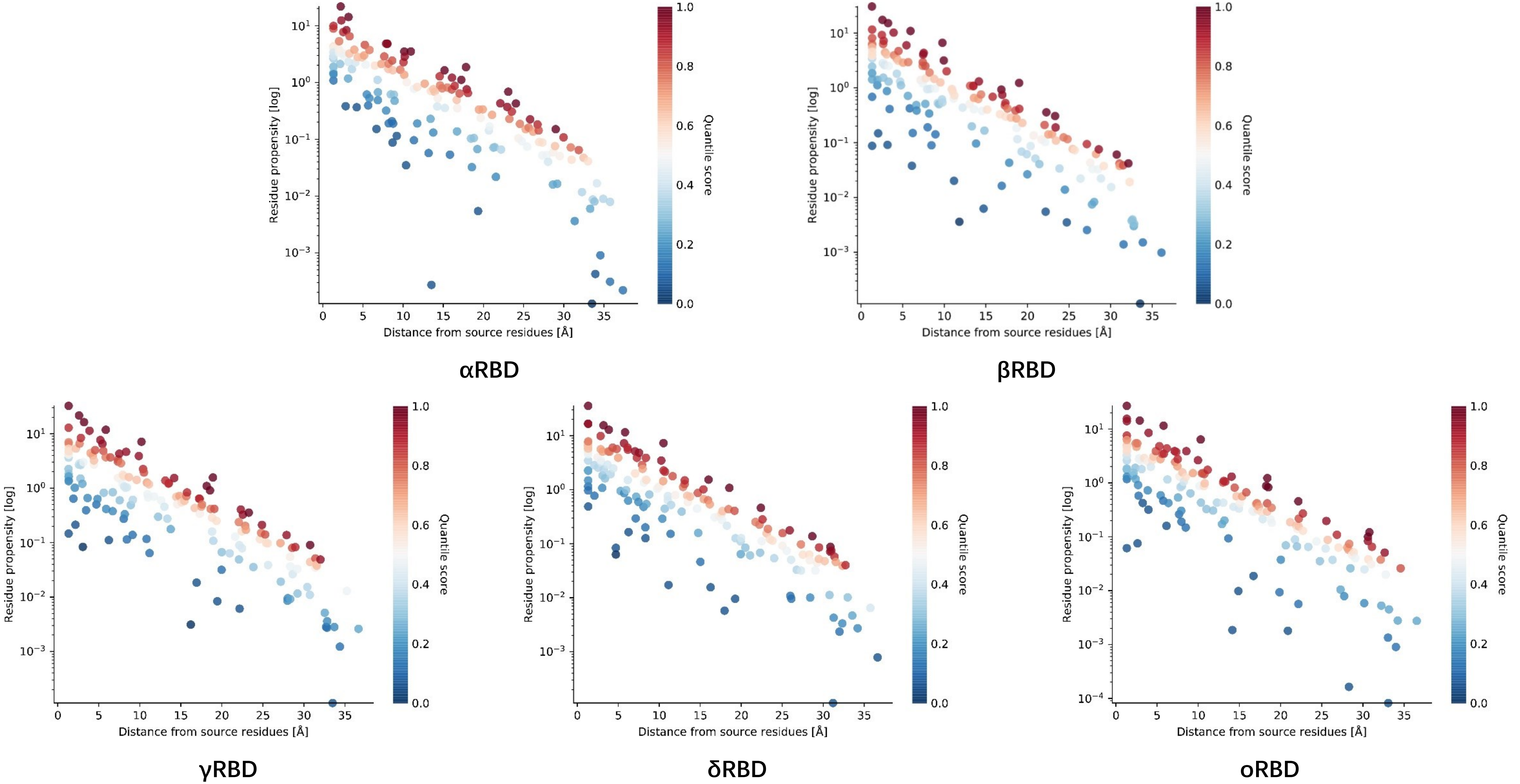

Figure S3. The hotspot view of high or low connectivity to the source sites located on the ACE2-binding region of various RBDs. All data points were plotted as propensity over distance from source, in which hotspots and coldspots are colored according to their quantile scores.

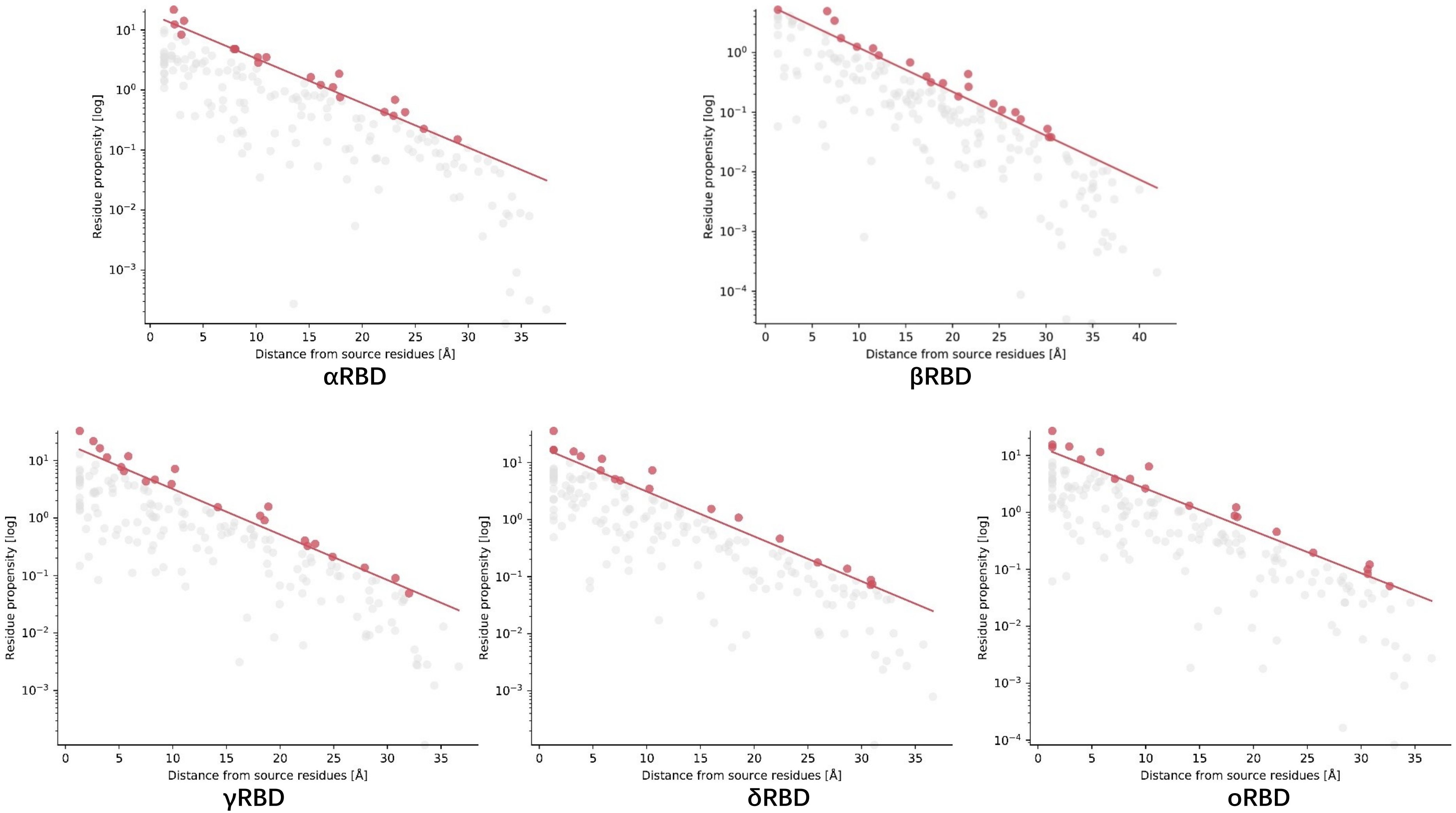

Figure S4. The view of residues relevant to ACE2-binding sites in various variant RBDs at 0.90 quantile score cut off.

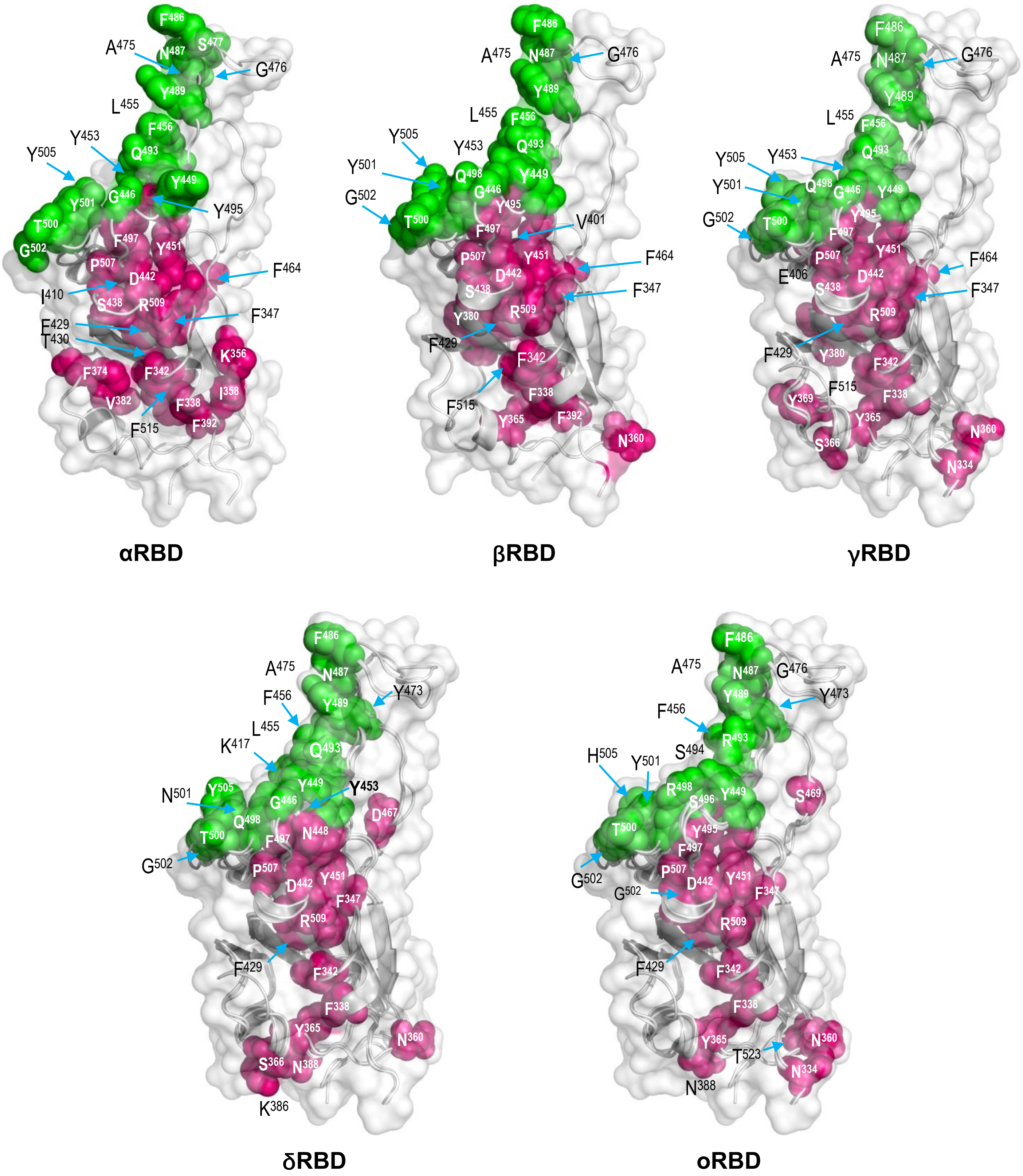

Figure S5. Mapping of relevant residues (hotpink) to the source residues (green) on various RBD structures. All the residues are labeled based on their structural coordinates (see Table S1 for their PDB entries).

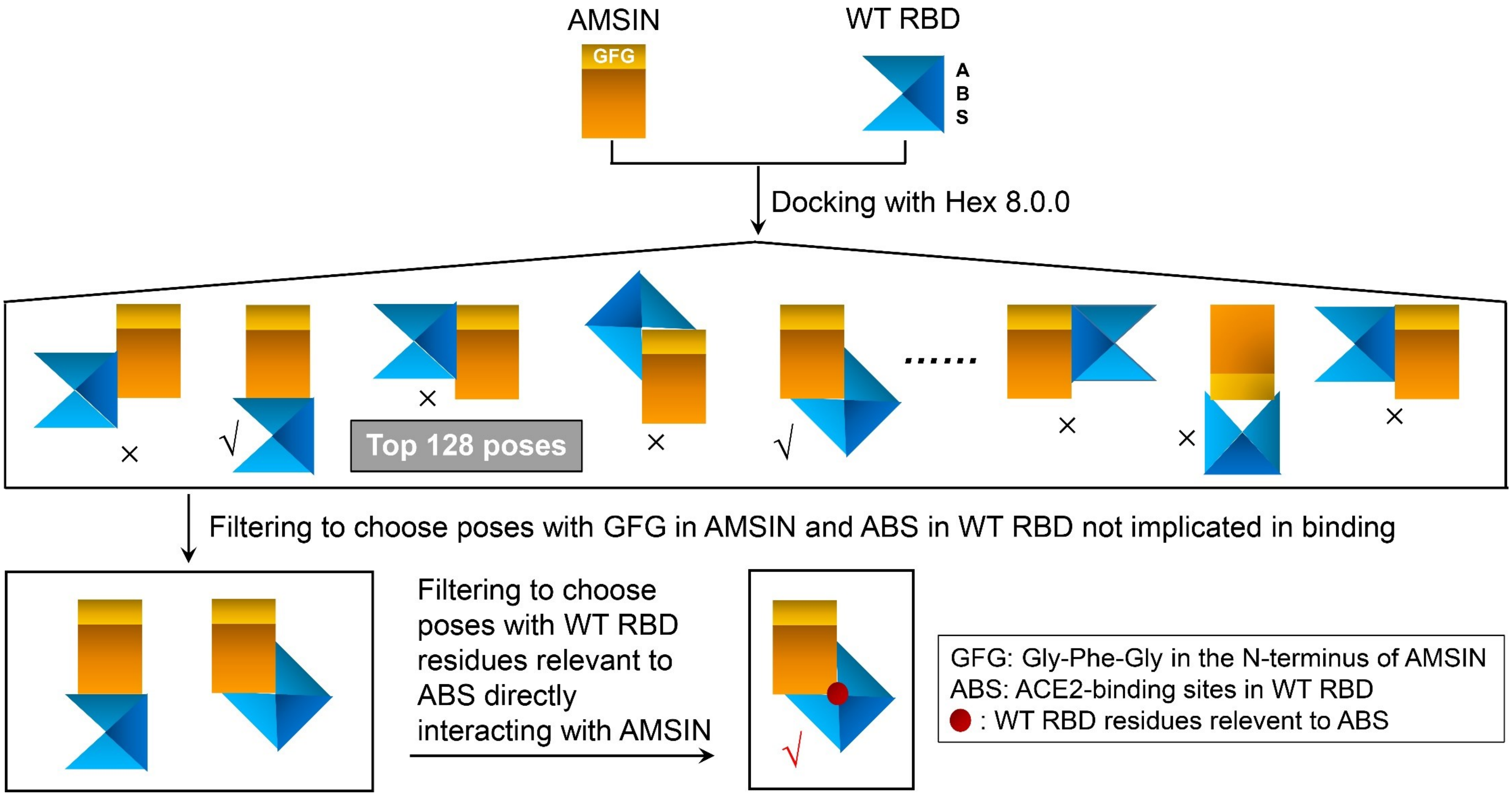

Figure S6. The strategy for choosing plausible computaional complexes between AMSIN and WT RBD. A set of top complexes (128 poses) are generated by protein docking using spherical polar fourier correlations with HEX based on shape complementation. Two rounds of filtering based on experimental and computaional data were carried out.

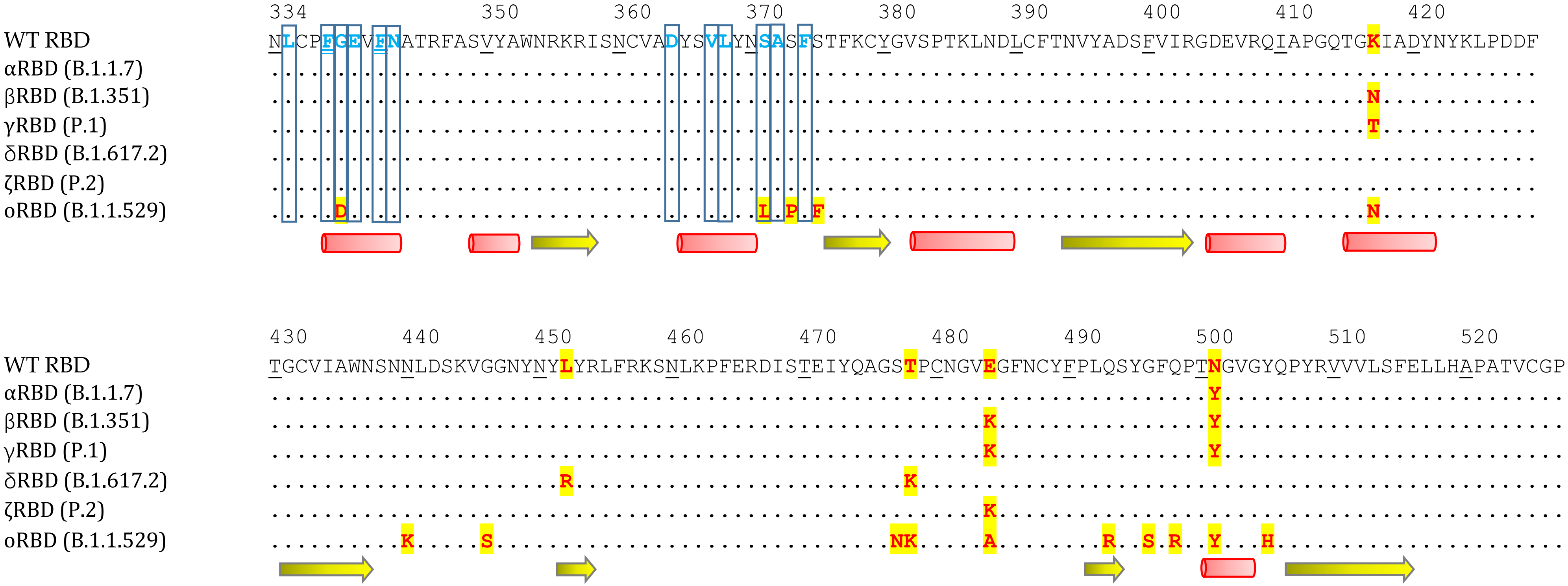

Figure S7. Multiple seqeunce alignment of WT RBD and ite variant RBDs. Identical residues to those of WT RBD are denoted by dots and nonidentical ones colored in red and shaded in yellow. Sites are numbered according to WT RBD. Secondary structure elements are extracted from the structure of WT RBD (pdb entry 6LZG). Residues predicted to interact with AMSIN are boxed and colored in cyan in WT RBD, in which two residues involved in direct AMSIN binding with its R^{26} are underlined twice. SARS-CoV-2 variant strains are shown in brackets.

		GenBank Number
AMSIN	GFGCPWNAYECDRHCVSK-GYTGGNC**R**GKIRQTCHC**Y**--	
Actinomyces	GFGCPWNAYECDRHCMSK-GYTGGNC**R**GKIRQTCHC**Y**--	WP_070658097.1
Stegodyphus mimosarum	GFGCPGNQYECNRHCRSN-GFTGGYC**K**GFLKMTCNC**Y**V-	KFM61041.1
Actinomyces	GFGCPFNAYQCHSHCLSI-GRRGGYC**R**GLVRQTCVC**Y**R-	WP_073451525.1
Actinomyces succiniciruminis	GFGCPFSERSCDTHCMTK-GYRGGYC**K**GAVRQTCVC**Y**K-	WP_210578363.1
Actinomyces wuliandei	PFGCPFNQYECHRHCRSA-GFRGGYC**K**GMFKQTCKC**Y**G-	WP_139738766.1
Actinomyces sp. 2119	PFGCPFNSFTCHRHCKSIPGYRGGYC**K**GRLNQTCKC**Y**R-	WP_119836392.1
Androctonus bicolor	GFGCPFNQGRCHRHCRSI-GRRGGYC**R**GIFKQTCAC**Y**RK	AIX87626.1
Ornithodoros papillipes	GFGCPFNQYECHAHCSGVPGYKGGYC**K**GLFKQTCNC**Y**--	ACJ04427.1
Ornithodoros turicata	GYGCPFNQYQCHSHCSGIRGYKGGYC**K**GLFKQTCTC**Y**--	QIG55621.1
Ornithodoros papillipes	GYGCPFNQYQCHSHCSGIRGYKGGYC**K**GTFKQTCKC**Y**--	ACJ04425.1
Alectorobius puertoricensis	GYGCPFNQYQCHSHCSGIRGYKGGYC**K**GTFKQTCKC**Y**--	ACJ04429.1
Ornithodoros rostratus	GYGCPFNQYQCHSHCSGIRGYKGGYC**K**GTFKQTCKC**Y**--	ACJ04428.1
Ornithodoros tartakovskyi	GYGCPFNQYQCHSHCSGIRGYKGGYC**K**GTFKQTCKC**Y**--	ACJ04431.1
Ornithodoros moubata	GYGCPFNQYQCHSHCSGIRGYKGGYC**K**GLFKQTCNC**Y**--	BAC10303.1
Ornithodoros savignyi	GYGCPFNQYQCHSHCKGIRGYKGGYC**K**GAFKQTCKC**Y**--	P0DV61.1

Figure S8. Multiple seqeunce alignment of representative defensins containing the dyad. The residues constituting the dyad are shown in red and boxed.
